# Analyzing the changing trend of corneal biomechanical properties under different influencing factors in T2DM patients

**DOI:** 10.1038/s41598-024-59005-7

**Published:** 2024-04-08

**Authors:** Juan Tang, Zhiwu Lin, Xingde Liu, Biao Li, Xiaoli Wu, Jing Lv, Xing Qi, Sheng Lin, Chuanqiang Dai, Tao Li

**Affiliations:** 1 Department of Endocrinology, Ziyang Central Hospital, Sichuan, China; 2 Department of Cardiothoracic Surgery, Ziyang Central Hospital, Sichuan, China; 3 Department of Ophthalmology, Ziyang Central Hospital, Sichuan, China; 4 Department of Orthopedics, Ziyang Central Hospital, Sichuan, China; 5 Department of Experimental Medicine, Ziyang Central Hospital, Sichuan, China

**Keywords:** T2DM, Corneal hysteresis, Corneal resistance factor, Ocular response analyzer, Diabetic keratopathy, Diseases, Endocrinology, Health care, Medical research, Risk factors

## Abstract

To analyze the changing trend of CH and CRF values under different influencing factors in T2DM patients. A total of 650 patients with T2DM were included. We discovered that the course of T2DM, smoking history, BMI, and FBG, DR, HbA1c, TC, TG, and LDL-C levels were common risk factors for T2DM, while HDL-C levels were a protective factor. Analyzing the CH and CRF values according to the course of diabetes, we discovered that as T2DM continued to persist, the values of CH and CRF gradually decreased. Moreover, with the increase in FBG levels and the accumulation of HbA1c, the values of CH and CRF gradually decreased. In addition, in patients with HbA1c (%) > 12, the values of CH and CRF decreased the most, falling by 1.85 ± 0.33 mmHg and 1.28 ± 0.69 mmHg, respectively. Compared with the non-DR group, the CH and CRF values gradually decreased in the mild-NPDR, moderate-NPDR, severe-NPDR and PDR groups, with the lowest CH and CRF values in the PDR group. In patients with T2DM, early measurement of corneal biomechanical properties to evaluate the change trend of CH and CRF values in different situations will help to identify and prevent diabetic keratopathy in a timely manner.

## Introduction

Diabetes is a common metabolic and endocrine disease caused by absolute or relatively insufficient insulin secretion. The basic pathological changes of diabetes are metabolic disorders that can lead to lesions in various systems and organs in the body^1,2^. The pathogenesis of diabetes includes pathophysiological processes, such as apoptosis, inflammation, neurotrophic damage and oxidative stress^3,4^. In the field of ophthalmology, there have been extensive studies on retinopathy, glaucoma and cataracts caused by diabetes^5–7^. In recent years, some scholars have reported that more than 70% of diabetes patients can develop diabetic keratopathy, and it has been confirmed that some patients can have morphological changes in the cornea^8–10^. Different from traditional diabetic retinopathy, glaucoma, cataracts and other diseases that easily lead to decreased vision as the main clinical symptoms, the clinical manifestations of diabetic keratopathy are often ignored. The main manifestations of these patients are decreased corneal sensation, delayed corneal epithelial regeneration, and bullous corneal endothelial disease, which is followed by severe lesions, such as persistent corneal epithelial erosion, superficial punctate keratopathy, and even corneal ulcers^11–13^. It is potentially a vision-threatening disease that can lead to decreased vision or permanent visual loss, especially after undergoing treatments such as phacoemulsification and vitrectomy^14–16^. Some scholars believe that long-term elevations in blood sugar levels may lead to impaired endothelial "pump" function before intraocular surgery^17^. The cornea is enriched in sensory nerve endings, so impaired glucose metabolism will inevitably lead to sensory impairment of the cornea. However, there is still a lack of objective evaluation methods for diabetic keratopathy^18^.

The cornea is located in the frontal segment of the eye, and its transparency and regularity play a significant role in providing approximately two-thirds of the refractive power of the eye^19,20^. In addition, the cornea is a mechanical barrier layer mainly composed of viscoelastic tissue, with certain stiffness and elasticity, and its integrity maintains the relatively constant intraocular pressure^21,22^. Therefore, corneal biomechanical properties, such as corneal hysteresis (CH) and corneal resistance factor (CRF), play important roles in maintaining eye integrity and corneal transparency. Moreover, CH predominantly reflects the viscous properties of the cornea, and CRF is a synthetic measure of corneal elastic properties^23,24^. Numerous studies have shown that corneal biomechanical properties are influenced by different factors, such as central corneal thickness (CCT), spherical equivalence (SE), corneal curvature (K), axial length (AL), intraocular pressure (IOP) and age^25,26^. Especially after various corneal diseases and corneal surgery, the deformation and pathological changes of the cornea may lead to changes in corneal biomechanics^27,28^. With the clinical application of the Ocular Response Analyzer (ORA) in ophthalmology, it is possible to evaluate biomechanical properties in living corneas^29,30^. Some research has reported that the measurement of corneal biomechanics by ORA has been widely used in the evaluation of corneal biomechanics of patients in the diagnosis and treatment of keratoconus, glaucoma and corneal refractive surgery^31–33^. Therefore, this study intended to use an ORA analyzer to measure the corneal biomechanics of T2DM patients and evaluate the influencing factors of corneal biomechanics in diabetes patients.

## Materials and methods

### Study patients

This was a retrospective study designed to investigate associations between corneal biomechanical properties and potential impact factors in T2DM patients. A total of 650 patients with T2DM who visited Ziyang Central Hospital from February 2022 to March 2023 were included. The measurements of CCT and AL were detected by an ultrasonic pachymeter (Sonomed Inc.1979 Marcus Avenue Lake Success, NY 11,042, USA). The IOP and SE were measured by Goldmans applanation tonometry and Corneal Topography (CSO, Firenze, Italy), respectively. Three measurements were taken in each eye. All eye data were collected from both eyes, and the average value was obtained.

**Inclusion criteria**: (1) symptoms of diabetes + plasma glucose level at any time ≥ 11.1 mmol/L (200 mg/dl); (2) fasting blood glucose (FBG) ≥ 7.0 mmol/L (126 mg/dl); (3) OGTT (Oral Glucose Tolerance Test) 2 h post meal plasma glucose ≥ 11.1 mmol/L; (4) the average CCT, IOP, SE and AL were 502.34 ± 12.48 µm, 12.73 ± 2.92 mmHg, 2.18 ± 0.91 D, and 24.68 ± 1.53 mm, respectively. (5) The CCT and Corneal curvature screening range were 490–515 µm and 42.87–44.56 D. Differences in CH and CRF caused by significant differences in CCT, IOP, SE, Corneal curvature, and AL were excluded.

**Exclusion criteria**: Keratoconus, suspected keratoconus, corneal dystrophy, glaucoma, acute renal injury, type 1 diabetes, diabetes ketoacidosis, acute hyperglycemia (accompanied by severe ketonuria), and acute infection.

### Clinical and laboratory data collection

The clinical data were obtained from medical records and included age, sex, course of T2D, hypertension, history of smoking, alcohol consumption, BMI, systolic blood pressure (SBP) and diastolic blood pressure (DBP). Laboratory parameters, such as the levels of total cholesterol (TC), triglycerides (TGs), high-density lipoprotein cholesterol (HDL-C), low-density lipoprotein cholesterol (LDL-C), hemoglobin (HbA1c), and fasting blood glucose (FBG), were measured in the morning following overnight fasting.

### Diagnosis and staging of DR

After excluding contraindications for fundus fluorescein angiography, all patients underwent this examination. Two experienced ophthalmologists from The First People’s Hospital of Ziyang staged the patient’s DR lesions based on fundus fluorescence angiography images according to the international clinical DR severity scale. The fundus manifestations in patients with diabetes were classified into DR stages: non-DR, mild-nonproliferative (mild-NPDR), moderate-nonproliferative (moderate-NPDR), severe-nonproliferative (severe-NPDR) and proliferative (PDR).

### Measurement of CH and CRF

The ORA measurement (Reichert Ophthalmic Instruments, Buffalo, New York, USA) is currently the main method for measuring the corneal biomechanics of CH and CRF. Based on the principle of dynamic bidirectional flattening, the ORA collected the P1 and P2 values during the two compression processes of the cornea. Then, the CH value was calculated using the numerical difference between the P1 and P2 values. The CH value mainly reflected the corneal viscosity. CRF was calculated through a specific formula that represented an indicator of evaluating corneal viscoelasticity. Both the CH and CRF values were provided directly by the ORA machine.

### Statistical analysis

Statistical management and analysis were conducted using SPSS 20.0 (SPSS Inc., Chicago, USA). Independent sample t tests were used to assess whether each parameter had a normal distribution. Categorical variables were compared using the X^2^ test. Logistic regression analysis was performed to evaluate factors associated with T2DM. Statistical significance was set at p < 0.05.

### Ethical committee acceptance code

This study adhered to the tenets of the Declaration of Helsinki and Malaysian Guidelines for Good Clinical Practice (GCP). This study protocol was reviewed and approved by the Ethics Committee of Ziyang Central Hospital (no. 202100951). A signed written informed consent was obtained from all patients prior to enrolment. The authors affirm that human research participants provided informed consent for the publication of their data.

## Result

We initially examined the medical records of 650 patients with T2DM. Among these patients, there were 348 males and 302 females, and the average age was 54.28 ± 18.27 years. The average course of T2DM (year) and the average BMI, FBG, SBP, DBP, HbA1c, TC, TG, HDL-C and LDL-C values of the study subjects were 58.29 ± 13.98 years, 26.28 ± 3.71 kg/m^2^, 8.74 ± 2.93 mmol/L, 147.23 ± 18.34 mmHg, 93.87 ± 8.02 mmHg, 8.58 ± 2.93%, 4.38 ± 1.38 mmol/L, 8.18 ± 1.52 mmol/L, 1.12 ± 0.29 mmol/L and 3.75 ± 1.07 mmol/L, respectively.

CCT and corneal curvature are the most important factors affecting CH and CRF^17,22^. Therefore, after excluding the effects of CCT and corneal curvature on CH and CRF, a total of 650 T2DM patients were included in this study. First, logistic linear regression analysis was performed to evaluate influencing factors associated with T2DM (Table 1). After adjusting for sex, age, course of T2DM, history of smoking, alcohol consumption, BMI, FBG, DR, SBP ≥ 130 mmHg, DBP > 90 mmHg and HbA1c, TC, TG, HDL-C, and LDL-C levels, T2DM was significantly associated with course of T2DM, history of smoking, BMI and FBG, DR, HbA1c, TC, TG, HDL-C, and LDL-C levels. These results showed that the course of T2DM, smoking history, BMI and FBG, DR, HbA1c, TC, TG and LDL-C levels were common risk factors for T2DM, while HDL-C levels were a protective factor. These differences were statistically significant (all P < 0.05).

**Table 1:** Logistic linear regression analysis of influencing factors related to T2DM.

Variable	OR (95%CI)	P value
Gender	1.23 (0.72, 1.56)	0.478
Age (year)	1.18 (0.78, 1.34)	0.281
course of T2DM (year)	1.89 (1.43, 2.46)	0.007
A history of smoking	1.57 (0.91, 1.92)	0.031
Alcohol consumption	0.92 (0.59, 1.45)	0.382
BMI	1.59 (1.12, 1.98)	0.002
FBG	1.72 (0.91, 2.34)	0.047
DR	2.07 (1.48, 2.91)	0.003
SBP ≥ 130 mmHg	1.42 (1.02, 2.37)	0.092
DBP > 90 mmHg	1.29 (0.93, 1.67)	0.185
HbA1c (%)	1.97 (1.21, 3.02)	0.003
TC (mmol/L)	1.71 (0.98, 2.35)	0.001
TG (mmol/L)	1.48 (0.65, 1.72)	0.032
HDL-C (mmol/L)	0.82 (0.43, 1.23)	0.021
LDL-C (mmol/L)	1.45 (0.78, 1.92)	0.006

T2DM, type 2 diabetes; BMI, body mass index; FBG, fasting blood glucose; DR, diabetic retinopathy; SBP, systolic blood pressure; DBP, diastolic blood pressure; TC, total cholesterol; TG, triglycerides; HDL-C, high-density lipoprotein cholesterol; LDL-C, low-density lipoprotein cholesterol. *p value < 0.05 was significant (logistic regression analysis).

To further explore the influence of the course of T2DM on corneal biomechanical properties, patients were divided into Group I (1 < T2DM course < 5 years), Group II (5 ≤ T2DM course < 10 years), Group III (10 ≤ T2DM course < 15 years), Group IV (15 ≤ T2DM course < 20 years) and Group V (T2DM course ≥ 20 years) according to the course of diabetes. As shown in Table 2, with the prolongation of the course of T2DM, the values of CH and CRF gradually decreased. LSD-t test analysis showed that the difference between CH and CRF values was statistically significant (P < 0.05).

**Table 2:** Biomechanical characteristics of cornea in T2DM patients with different diabetes course.

Course of T2DM (year)	CH (mmHg)		CRF (mmHg)	
	Median	95% CI	Median	95% CI
Group I (n = 145)	12.12	11.02, 12.34	12.45	11.32, 13.48
Group II (n = 104)	11.68	10.21, 12.08	11.92	10.82, 12.34
Group III (n = 115)	11.24	9.34, 11.23	11.44	10.93, 12.03
Group IV (n = 109)	10.79	9.02,11.08	10.92	9.78,11.73
Group V (n = 177)	10.38	8.91, 10.92	10.33	9.67, 11.21
P值	0.001		0.041	

Group I(1 < T2DM course < 5), Group II (5 ≤ T2DM course < 10), Group III (10 ≤ T2DM course < 15) , Group IV (15 ≤ T2DM course < 20) and Group V (T2DM course ≥ 20).

CH Corneal hysteresis, CRF Corneal resistance factor.

Then, we divided these subjects into the FBG1 group (7.0 mmol/L < FBG < 9 mmol/L), FBG2 group (9.0 mmol/L ≤ FBG < 11 mmol/L), FBG3 group (11 mmol/L ≤ FBG < 13 mmol/L), and FBG4 group (FBG ≥ 13 mmol/L) based on FBG values. As expected, the values of CH and CRF in T2DM patients gradually decreased as FBG levels increased (Table 3). Furthermore, we grouped these subjects regarding to HbA1c levels as Group A (6.5 < HbA1c (%) ≤ 8), Group B (8 < HbA1c (%) ≤ 10), Group C (10 < HbA1c (%) ≤ 12), and Group D (HbA1c (%) > 12). The results showed that in T2DM patients, with the increase in HbA1c (%), the values of CH and CRF gradually decreased, especially when HbA1c (%) > 12, and the values of CH (1.85 ± 0.33 mmHg) and CRF (1.28 ± 0.69 mmHg) decreased the most (Table 4).

**Table 3:** Biomechanical characteristics of cornea in T2DM patients with different fasting blood glucose.

Group	FBG1 (n = 137)	FBG2 (n = 165)	FBG3 (n = 158)	FBG4 (n = 190)	F	P
CH (mm Hg)	11.83 ± 0.79*	11.42 ± 0.92*	10.98 ± 0.77*	10.45 ± 0.82*	72.32	0.021
CRF (mm Hg)	12.32 ± 1.36*	11.91 ± 1.08*	11.24 ± 1.13*	10.78 ± 0.89*	83.23	0.003

FBG1 group(7.0 mmol/L < FBG < 9 mmol/L), FBG2 group(9.0 mmol/L ≤ FBG < 11 mmol/L), FBG3 group(11 mmol/L ≤ FBG < 13 mmol/L), and FBG4 group (FBG ≥ 13 mmol/L).

CH Corneal hysteresis, CRF Corneal resistance factor.

*Comparision among the four groups each other LSD-t P < 0.05.

**Table 4:** Biomechanical characteristics of cornea in T2DM patients under different HbA1c (%) conditions.

HbA1c (%)	6.5–8 (n = 142)	8–10 (n = 173)	10–12 (n = 189)	> 12 (n = 146)	F	P
CH (mm Hg)	12.25 ± 0.87*	11.87 ± 0.97*	11.14 ± 0.82*	10.78 ± 0.91*	58.23	0.001
CRF (mm Hg)	12.17 ± 1.18*	11.82 ± 1.01*	11.24 ± 1.04*	10.92 ± 0.72*	76.29	0.037

CH Corneal hysteresis, CRF Corneal resistance factor.

*Comparision among the groups each other LSD-t P < 0.05.

Logistic linear regression analysis showed that DR was an independent risk factor for T2DM. Therefore, fundus fluorescence angiography examination was performed on patients. There were 207 patients in the non-DR group, 145 patients in the mild-NPDR group, 167 patients in the moderate-NPDR group, 89 patients in the severe-NPDR group, and 42 patients in the PDR group. Further statistical analysis of the mean changes in CH and CRF in each group revealed that compared to that in the non-DR group, the CH and CRF values in the mild-NPDR, moderate-NPDR, severe-NPDR, and PDR groups gradually decreased, with the lowest CH and CRF values in the PDR group (Table 5).

**Table 5:** Analysis of corneal biomechanical properties under different DR stages.

Group	Non-DR	Mild-NPDR	Moderate-NPDR	Severe-NPDR	PDR	F	P
CH (mm Hg)	12.21 ± 0.89*	11.82 ± 0.72*	11.31 ± 0.91*	10.94 ± 0.91*	10.38 ± 0.91*	82.31	0.016
CRF (mm Hg)	12.39 ± 0.92*	11.87 ± 1.04*	11.19 ± 0.94*	10.78 ± 0.87*	10.28 ± 0.85*	77.84	0.003

CH Corneal hysteresis, CRF Corneal resistance factor, NPDR Non proliferative diabetic retinopathy, PDR proliferative diabetic retinopathy.

*Comparision among the groups each other LSD-t P < 0.05.

## Discussion

The cornea is located at the outermost layer of the eyeball and has viscoelastic properties. The biomechanical properties of the collagen fibers existing in its stromal layers play an important role in maintaining the inherent morphology of the eyeball and protecting its inner structure^34^. Related studies have shown that corneal biomechanical properties are closely related to factors such as corneal curvature, corneal thickness, and refractive index. Therefore, it was initially applied to exclude keratoconus in corneal refractive surgery^35,36^. In recent years, with the application of the ORA analyzer in corneal biomechanical measurements of patients with cataracts, glaucoma, and other diseases, research has reported that patients with primary open angle glaucoma may experience changes in corneal biomechanical parameters, and the corneal biomechanical analyzer contributed to the early diagnosis and evaluation of primary open angle glaucoma^37^. Moreover, Giacomo reported that both coaxial microincision Phaco and standard incision Phaco can alter corneal biomechanical characteristics. Additionally, coaxial microincision Phaco recovered corneal biomechanical characteristics faster than standard incision Phaco, which has important reference significance for selecting cataract surgery incisions^38^. ORA is noncontact eye response analyzer that made it possible to evaluate the biomechanical properties of living corneas. The main indicators of ORA are CH and CRF. CH represents the corneal viscous resistance, which is the ability to absorb and disperse energy. CRF represents the overall stiffness of the cornea, reflecting the cumulative effect of resistance when the cornea is compressed and deformed by airflow. This process is greatly affected by the hydration of the cornea. Many studies have shown that the oxidative stress reaction in the corneal stroma of patients with diabetes is greatly affected by blood sugar, and patients often have hydration dysfunction, which further leads to corneal neurotrophic disorder^8,39^.

In this study, we found that the course of T2DM and FBG, DR, and HbA1c levels were risk factors for T2DM by logistic linear regression analysis. Therefore, we further statistically analyzed the CH and CRF values under different states of the T2DM course and FBG, DR, and HbA1c levels. Interestingly, after excluding the effects of factors such as corneal thickness and curvature on CH and CRF, the results showed that as the course of T2DM was prolonged, FBG increased, DR staging worsened, HbA1c accumulated, and CH and CRF values gradually decreased. The above results indicated that with the deterioration of the general condition in T2DM patients, the corneal biomechanics of patients gradually decreased. Markoulli reported that hyperglycemia had important effects on the morphology, physiological function, metabolism, and other aspects of corneal cells^40^. Especially in the condition of hyperglycemia, the abnormal expression of collagen fibers (type I and III collagen fibers) or protein peptides (such as keratin glycan, membrane glycan, or core proteoglycan) in the corneal stroma layer further affects the normal cross-linking between collagen fibers. This leads to changes in the number and structure of existing fibers in the corneal stroma layer, thereby affecting corneal edema and reducing the visual quality of patients. Furthermore, Chang found that corneal epithelial basal cells originated from corneal limbal stem cells in T2DM patients and he also discovered that the density of corneal epithelial basal cells decreased, while the structure and space of cells became wider^41^. As blood sugar levels increase, laser confocal microscopy further showed that the arrangement of corneal fibrous tissue in the corneal stromal layer was loose, and the density of collagen fibers decreased. In addition, Di G considered that the size and nucleus of corneal stroma cells in patients with diabetes were large, the extracellular collagen fibers were uneven in thickness and disorderly arranged, the number of rough endoplasmic reticulum, mitochondria, Golgi bodies and other organelles were fewer, and the basement membrane was thin and discontinuous, indicating that the corneal cells in diabetes patients had low metabolism and inactive proliferation^42^.

In clinical work, retinal angiography for diabetes patients is restricted by allergies, renal function, liver function and other factors. Moreover, mydriasis examination takes a long time, so not all patients with diabetes are willing to undergo fundus examination. In this study, we discovered that as the DR lesion worsened, the CH and CRF values gradually decreased. Therefore, we should pay attention to the decrease in the biomechanical performance of T2DM. Matlock demonstrated that the cornea was one of the organs with the widest distribution of nerve endings in the body, and corneal nerve fibers play an important role in maintaining the homeostasis of the corneal epithelium^43^. Cruzat showed that damaged corneal nerve fibers could lead to the disordered arrangement of corneal fiber structure and tissue edema, thus further leading to a decrease in corneal biomechanics^44^. Moreover, the loss of corneal nerves could lead to a decrease in the supply of neurotrophin, and patients would suffer from corneal epithelial erosion and ulcer formation, which could result in eye redness, photophobia, tears and other discomfort^45^. In this study, we discovered that the course of T2DM, smoking history, BMI and FBG, DR, HbA1c, TC, TG and LDL-C levels were common risk factors for T2DM, and as the course of T2DM was prolonged, FBG levels increased, DR staging worsened, HbA1c accumulated, and CH and CRF values gradually decreased. These results are different from those of other studies^46,47^. Scholars, such as Ramm, reported that compared with healthy subjects, CH and CRF values were increased in T2DM patients^46^. However, the above study involved a small sample size and did not strictly control for factors such as CCT, SE, AL and IOP. Related studies have shown that hyperglycemia could cause corneal stroma edema and further lead to an increase in corneal thickness, thus leading to an increase in CH and CRF measurement values. In this study, we excluded the influence of CCT, SE, AL and IOP on CH and CRF, and the CH and CRF values were collected as the average values of patients before and after blood sugar treatment. Furthermore, we believe the CH and CRF values measured under hyperglycemia were only increased due to corneal edema. After controlling for the influence of the patient’s blood sugar on corneal edema, the true corneal biomechanics of the T2DM patient were reduced. Therefore, our study suggests that patients with diabetes should be examined for corneal biomechanical properties, especially patients with a long course of diabetes and unstable blood sugar control. This is instructive for the early evaluation of corneal neuropathy in patients with type 2 diabetes and is of great significance for early screening and preventing DR progression. Moreover, our study proposes that the corresponding CH and CR values should be compared and analyzed according to the course of T2DM, FBG levels, HbA1c levels and DR staging in clinical work.

The major limitations of this study were that it was a retrospective study, which was worth further exploration.

## Conclusion

In summary, our study investigated the association between corneal biomechanical properties measured with the ORA in T2DM patients and potential impact factors that we mentioned after excluding the influence of CCT and corneal curvature. We discovered that as the course of T2DM was prolonged, FBG levels increased, DR staging worsened, HbA1c accumulated, and CH and CRF values gradually decreased. Moreover, our study included the corresponding CH and CRF data for clinical reference under different states of diabetes. Based on our findings, our study proposed that we should compare and analyze the corresponding CH and CR values according to the course of T2DM, FBG levels, HbA1c levels and DR staging in clinical work.

## Data Availability

All data generated or analysed during this study are included in this published article. The datasets used and/or analysed during the current study available from the corresponding author on reasonable request.

## Abbreviations

CH: Corneal hysteresis
CRF: Corneal resistance factor
CCT: Central corneal thickness
T2DM: Type 2 diabetes
BMI: Body mass index
FBG: Fasting blood glucose
DR: Diabetic retinopathy
SBP: Systolic blood pressure
DBP: Diastolic blood pressure
TC: Total cholesterol
TG: Triglycerides
HDL-C: High-density lipoprotein cholesterol
LDL: Low-density lipoprotein cholesterol
